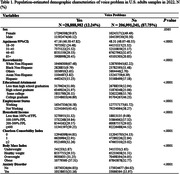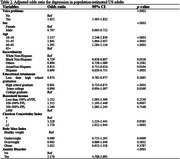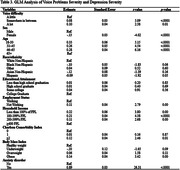# Voice Problems and Depression: Findings from the 2022 National Health Interview Survey

**DOI:** 10.1002/alz.088615

**Published:** 2025-01-03

**Authors:** Ickpyo Hong, HOKYUNG LEE

**Affiliations:** ^1^ Yonsei University, Wonju, Gangwon‐do Korea, Republic of (South); ^2^ Yonsei University, 원주, Gangwon-do Korea, Republic of (South)

## Abstract

**Background:**

In contemporary society, essential for quality of life and professional success, is impacted by voice problems affecting 3∼9% of the population. These issues, leading to significant health and economic burdens, can alter voice frequency and tone, impacting self‐esteem, social interactions, and professional outcomes. Voice problems are linked to social isolation and depression, a major emotional disorder causing lethargy and loss of hope. Studies, including the 2012 National Health Interview Survey, show higher depression rates in individuals with voice issues. Our study aimed to use the most recent national survey data and examine this link, and also provide insights for better treatment strategies.

**Method:**

This study utilized the 2022 National Health Interview Survey to explore the relationship between voice problems and depression in U.S. adults. The independent variable is self‐reported voice problems, while the dependent variable is depression, measured using the PHQ‐8 questionnaire. Covariates included sex, age, race, education, income, comorbidities, body weight, and anxiety disorders. Propensity score matching techniques and multivariate logistic regression were used to control for selection bias between the comparison groups. Subgroup analysis investigated the link between the severity of voice problems and depression.

**Result:**

The study found that 12.24% of U.S. adults reported voice problems in 2022. Demographics and health conditions significantly differed between those with and without voice problems. Depression was 1.6 times more likely in individuals with voice problems, and lower in men and non‐white ethnicities (OR = 1.655, 95% CI [1.495, 1.832]). After propensity score matching, covariates were balanced, confirming a consistent, significant association between voice problems and depression across various adjustment models. Subgroup analysis showed a stronger association of depression severity with voice problems severity and anxiety disorders, with women exhibiting a negative correlation(β = 0.89, p <.0001; β = ‐0.13, p <.0001).

**Conclusion:**

The study highlights a strong association between voice problems and increased depression risk, especially in women and those with anxiety disorders. These findings emphasize the importance of specialized intervention strategies to address these issues.